# Effect of visit length and a clinical decision support tool on abdominal aortic aneurysm screening rates in a primary care practice

**DOI:** 10.1111/j.1365-2753.2010.01625.x

**Published:** 2012-06

**Authors:** John Eaton, Darcy Reed, Kurt B Angstman, Kris Thomas, Frederick North, Robert Stroebel, Sidna M Tulledge-Scheitel, Rajeev Chaudhry

**Affiliations:** 1 Internal Medicine Resident, Department of Internal Medicine, Mayo Clinic College of MedicineRochester, MN, USA; 2 Assistant Professor of Medicine, Division of Primary Care Internal Medicine, Mayo Clinic College of MedicineRochester, MN, USA; 3 Assistant Professor of Family Medicine, Department of Family Medicine, Mayo Clinic College of MedicineRochester, MN, USA; 4 Assistant Professor of Medicine, Division of Primary Care Internal Medicine, Mayo Clinic College of Medicine, Rochester, MN, USA and Assistant Professor of Medicine, Global Products and Services, Mayo Clinic RochesterRochester, MN, USA; 5 Assistant Professor of Medicine, Division of Primary Care Internal Medicine, Mayo Clinic College of Medicine, Rochester, MN, USA and Assistant Professor of Medicine, Center for Innovation, Mayo Clinic College of MedicineRochester, MN, USA

**Keywords:** abdominal aortic aneurysm, clinical decision support systems, delivery of health care, patient care team, patient-centred care, preventive services

## Abstract

**Rationale, aims and objectives:**

In 2005, the US Preventive Services Task Force issued recommendations for one-time abdominal aortic aneurysm (AAA) screening using abdominal ultrasonography in men aged 65 to 75 years with a history of smoking. However, despite a mortality rate of up to 80% for ruptured AAAs, providers order the screening for a minority of patients. We examined AAA screening rates among providers and investigated the role of visit duration and other factors in whether patients received screening. We also looked for potential interventions to improve compliance.

**Methods:**

We retrospectively reviewed the records of patients who visited our clinic over a 4-month period and met the US Preventive Services Task Force criteria for AAA screening when our practice had a real-time decision support tool implemented to identify patients due for the screening. We also surveyed our clinic's providers about their knowledge and attitudes regarding AAA screening.

**Results:**

Despite the use of physician reminders, providers ordered screening for only 12.9% of eligible patients. Screening was more likely to be ordered during longer visits versus shorter ones (24% vs. 6%). When surveyed, most providers (70.6%) indicated that a nurse-directed ordering system would improve compliance.

**Conclusions:**

This study illustrates that physician reminders alone are not sufficient to improve care and that more time is needed for preventive services. This provides additional support for the use of a multidisciplinary approach to preventive screening, as in a patient-centred medical home. In a patient-centred medical home, a care team of physicians, nurses and office staff use technology such as clinical decision support to provide comprehensive, coordinated patient care.

## Introduction

An abdominal aortic aneurysm (AAA) is a dilation of the aorta below the renal arteries greater than 3.0 cm. AAAs occur in up to 10% of men and 1% of women aged 65 to 79 years [1, 2]. In addition to male gender, other risk factors for AAA are older age, current or past tobacco use and a family history of AAA [3]. Mortality after aneurysm rupture is 80% for patients who reach the hospital and 50% for those who undergo emergency surgical repair [4]. About 2% of aneurysms less than 4 cm rupture within 5 years, while 25% of aneurysms greater than 5 cm rupture in this period [3]. Prior to the adoption of screening practices, 9000 AAA deaths occurred in the USA annually, most of them in men older than 65 years [4].

In a meta-analysis of four population-based studies, screening in men 65 years or older was shown to significantly reduce AAA mortality [4]. This mortality benefit extends beyond the initial period of the screening. Seven years after screening, the reduction in AAA mortality and all-cause mortality was sustained. In addition, the cost-effectiveness based on the 7-year follow up was estimated at $19 500 per life-year gained [5]. If an AAA is absent on screening ultrasound, only 1% of 65-year-old men experience one in the next 5 years [6].

Responding to this evidence, the US Preventive Services Task Force (USPSTF) issued recommendations in 2005 for one-time AAA screening using abdominal ultrasonography in men aged 65 to 75 years who had a history of smoking [7]. Despite these recommendations, many patients do not receive appropriate screening for this condition [8]. Our goal was to examine AAA screening ordering practices in an academic primary care internal medicine clinic that uses physician reminders based on real-time clinical decision support for preventive screening and to identify why screening ordering rates vary among providers. We also explored providers' knowledge of AAA screening guidelines and their perceptions of current screening practices in their clinic. Finally, we looked for opportunities to improve AAA screening compliance.

## Methods

### Study design and participants

We retrospectively reviewed the records of all men aged 65 to 75 years with outpatient visits in the primary care practice of Mayo Clinic Rochester from 1 January to 30 April 2008, who met the USPSTF guidelines for AAA screening [7] and were current or former smokers. Men with a prior diagnosis of AAA and those who had already had an abdominal ultrasound or computed tomography scan for a reason other than AAA screening within the past 5 years were excluded. We abstracted these variables for the first visit during the study interval: appointment date, appointment type (general medical examination vs. other visit type), provider role (staff physician vs. other provider), provider gender and whether AAA screening ultrasound was ordered during the visit.

General medical examinations were at least 40-minute visits in which health maintenance was typically discussed. Other examination types included acute-care visits and follow-up examinations, which were typically shorter. Other providers included residents and midlevel providers. For patients for whom AAA screening was ordered, we recorded whether it was completed. In addition, during the 4-month study period, we counted how many outpatient visits occurred before the test was ordered. For example, a 65-year-old male smoker who fit the USPSTF criteria for screening would be assigned a 0 if AAA screening had been ordered the first time he had visited the clinic since turning 65; a 75-year-old man who met AAA screening criteria would be assigned a 4 if he had been seen four times since the guidelines were in place before AAA screening was ordered. For patients for whom screening was not ordered, we examined whether the rationale for not ordering screening was documented; if so, we recorded the specific rationale.

### Survey of providers

We developed a seven-item, anonymous electronic survey to assess providers' knowledge of AAA screening guidelines, their perceptions of current screening practices in their clinic, their opinions regarding barriers to screening and interventions to improve rates of screening. Depending on the question, the survey asked respondents to select either the single best answer or multiple answers. We emailed the survey to all 237 health care providers in the primary care internal medicine clinic of Mayo Clinic Rochester.

### Data analysis

We analysed the data using JMP software 7.0.1 (SAS Institute, Cary, NC, USA). Categorical variables were summarized as frequencies and percentages. A *P*-value of <0.05 was considered statistically significant. Univariate and multivariate logistic regression models used AAA screening ultrasound test ordered (yes vs. no) as the outcome variable. Independent variables examined were appointment type (general medical examination vs. other), provider role (staff physician vs. other) and provider gender. This study was approved by Mayo Clinic Rochester's Institutional Review Board.

## Results

### AAA screening ordering performance

We identified 442 eligible patients during the study period. Of the 442, 57 (12.9%) patients had an ultrasound ordered to screen for AAA. All 57 completed the test (95% confidence interval [CI]: 0.9–1.0). Of the 442 patients, 164 (37.1%) were seen for a general medical examination, and 278 (62.9%) were seen for another type of appointment (e.g. acute-care visit, follow-up examination). Most patients (340, 76.9%) were seen by staff physicians, while the remaining 102 (23.1%) were seen by another provider (64 by residents, 16 by midlevel providers and 22 by registered nurses).

Most patients (340, 76.9%) were seen by male providers. Although AAA screening was not ordered for 385 (87.1%) eligible patients, the rationale for not ordering AAA screening was documented for just six patients (1.3%). For three of these six patients, the rationale was patient refusal. For the remaining three, the provider documented that the patient was not a current smoker and therefore did not meet AAA screening eligibility criteria. For all three of these patients, our records indicated that the patient was a former smoker, suggesting that the provider was either unaware that the patient had smoked previously or was not fully familiar with the USPSTF screening guidelines.

Abdominal aortic aneurysm screening for eligible patients was ordered during 24% of general medical examinations, while screening was ordered during just 6% of other appointment types. Fourteen per cent of staff physician appointments and 9% of non-staff physician appointments resulted in AAA screening ordering. In addition, AAA screening was ordered during 13% of appointments with male providers and 12% of appointments with female providers. No statistically significant differences in AAA screening ordering practices were observed between staff physicians and other providers or between male and female providers (Table 1). In a multivariate regression analysis adjusted for provider type and gender, patients seen during a general medical examination were significantly more likely to have AAA screening ordered than those seen during other appointment types (OR, 4.5 [95% CI: 2.5–8.4], *P* < 0.0001). For the 57 patients who had AAA screening ordered, the mean number of eligible visits prior to having the screening ordered was 4.1 (95% CI: 3.4–4.8), with a range of 1 to 11. Only 13 (2.9%) of the 442 patients had the screening ordered at the first eligible visit.

**Table 1:** Effects of appointment type and provider role or gender on ordering rates of abdominal aortic aneurysm screening in a primary care internal medicine clinic

Logistic regression analysis				

	Univariate		Multivariate	
		
Variable	OR (95% CI)	*P*-value	OR (95% CI)	*P*-value
Appointment type (general medical exam vs. other exam)	4.5 (2.5–8.4)	<0.0001	4.5 (2.5–8.4)	<0.0001
Provider gender (male vs. female)	1.1 (0.4–1.8)	0.69	0.8 (0.4–1.8)	0.59
Provider type (staff physician vs. other)	1.7 (0.8–3.8)	0.15	1.6 (0.7–3.7)	0.26

CI, confidence interval; OR, odds ratio.

### Provider knowledge and attitudes regarding AAA screening

A total of 109 of 237 providers responded to the electronic survey, for a 46.0% response rate. Provider perceptions of their own knowledge of AAA screening guidelines varied. Only 19.3% of providers felt that they were very familiar with the guidelines, whereas 46.8% identified themselves as somewhat familiar, and 33.9% identified themselves as not familiar (Table 2). However, 65.1% of the respondents identified the correct frequency of screening (once in a lifetime, assuming that the initial test is normal), and 86.7% recognized men as the appropriate gender for screening. Furthermore, 74.3% identified current smokers as eligible, and 79.0% identified former smokers as eligible. Slightly more than half of the providers (58.1%) selected the appropriate age range for screening (65 to 75 years) (Table 2).

**Table 2:** Primary care internal medicine clinic providers' perceptions and knowledge regarding abdominal aortic aneurysm (AAA) screening guidelines and practices*

Question	Possible response	% response
1. Familiarity with US Preventive Services Task Force AAA screening guidelines	Very	19.3
	Somewhat	46.8
	Not	33.9
2. Identify correct frequency of AAA screening	Once in a lifetime	65.1
3. Identify correct specific AAA screening criteria	Men	86.7
	Current smokers	74.3
	Former smokers	79.0
	Age 65 to 75 years	58.1
4. Estimate what percentage of your eligible patients have undergone the recommended AAA screening	<25	34.6
	25 to 49	22.4
	50 to 75	23.4
	>75	5.6
	Not applicable	14.0
5. Estimate what percentage of eligible patients in the primary care internal medicine clinic have undergone the recommended AAA screening	<25	49.1
	25 to 49	38.2
	50 to 75	12.7
	>75	0.0
6. Identify factors that may contribute to poor AAA screening ordering practices in the primary care internal medicine clinic	Lack of provider knowledge	64.2
	Lack of patient knowledge	45.3
	Forgot to order	48.1
	Physician-dependent ordering system	41.5
7. Do you believe a nurse-driven ordering protocol would improve AAA screening ordering rates?	Yes	70.6
	Unsure	20.2
	No	9.2

* A total of 109 respondents: residents, 42.2%; nurses, 30.3%; staff, 22.0%; midlevel providers, 2.8%; not applicable, 2.7%.

When asked about their personal AAA screening ordering practices, most providers (80.4%) estimated that fewer than 75% of their eligible patients had undergone the recommended screening (Table 2). When asked to estimate the percentage of eligible patients in the clinic as a whole who had undergone the screening, 49.0% of providers estimated that fewer than 25% of clinic patients had, and none of the providers believed that more than 75% of patients had been appropriately screened.

Many respondents (64.2%) thought that a lack of provider knowledge contributed to poor AAA screening practices. Almost half of the respondents (48.1%) reported that forgetting to issue the order led to screening failures; 45.3% believed a lack of patient knowledge about screening recommendations was also a contributing factor. Some respondents (41.5%) thought that a physician-dependent ordering system played a role in the low compliance. Most respondents (70.6%) believed that a nurse-directed protocol for ordering AAA screening would improve compliance (Table 2).

## Discussion

This study illustrates that physician reminders alone are insufficient for providing the recommended AAA screening to eligible patients. Providers ordered AAA screening for fewer than 15% of patients who met the recommended USPSTF screening criteria. It was rare for screening to occur on the first visit, resulting in many missed opportunities (*n* = 4.1 visits) to screen. The longer visit was an independent predictor of screening for AAA. A provider survey identified factors that may contribute to the low screening rates and suggested potential solutions, chiefly the incorporation of a multidisciplinary approach to preventive care administration.

### Screening rates and visit time

With an increasing number of recommended screening tests coupled with shorter visit times, there is a higher burden on the physician to recall appropriate screening recommendations and remember to order them. It has been estimated that 1773 hours of a physician's annual time is spent on preventive services [9]. Consequently, lack of time has been identified as an important barrier in delivering preventive care [10,11]. Our study provides further evidence that visit time is an important determinant for preventive screening. Patients were more likely to have an AAA screening ultrasound ordered during a longer general medical examination, which usually has more time allotted (40 minutes) and often has a disease-prevention component. During longer medical examinations, 24% of eligible patients had the recommended AAA screening ordered, compared with only 6% during shorter visits.

Once the provider placed the order, patient follow-through with test completion was 100%. Our results showed that the test itself was not a barrier to completion. This contrasts sharply with colonoscopy screening, in which completion rates approximate 50% [12]. On average, the number of missed opportunities (visits) that occurred for patients for whom screening was ordered was 4.1. The burden of time per visit and preventive screening was illustrated in our survey, in which providers identified that forgetting to order the test and a physician-dependent ordering system were important factors in low screening rates.

### Physician reminders to order screening

The primary care clinic examined in this study uses health information technology (HIT), specifically real-time clinical decision support, to identify patients who are due for the screening, reminding providers to order age- and gender-specific screening tests and tests for chronic condition management. This protocol accurately identifies patients in need of screening tests, including AAA screening, at each office visit.

Paper copies of the clinical decision support tool summary screen of tests needed for each patient are placed outside the examination room door as well as in the patient's hands once the patient is taken to the room. However, the provider must still review the recommendation during the visit, order the test and explain it to the patient. Prior to implementation of this system, our AAA screening rates were only 3%. In general, our findings support other studies that have shown that provider-reminder systems have only a modest impact on screening practices. In a systemic review of the various types of physician prompts, including combined paper and computer reminders, paper-only reminders and computer-only reminders, the delivery of preventive care was improved by an average of only 12% to 14% [13]. As such, practice redesign is also needed so that all members of the care team, including the physician, work together to improve compliance.

### Provider-related reasons for failure to order screening

We identified some provider-related reasons for low rates of AAA screening ordering. First, our survey showed that providers have not yet internalized the recent guidelines. In fact, only a minority felt that they were very familiar with USPSTF guidelines for AAA screening, and their ability to identify the specific components of the guidelines varied greatly. Second, a large proportion of providers reported that they forgot to address the screening during the visit. Considering the once-a-lifetime recommended screening for AAA and the other competing screening tests that need completion at more frequent intervals (e.g. cholesterol levels, mammography, colonoscopy), it is understandable that AAA screening may not be foremost in the minds of providers – even during medical examinations in which prevention is stressed.

Provider perceptions about their personal screening ordering practices versus the clinic's practices as a whole were similar in that many felt that fewer than 25% of their own eligible patients and those of other providers had undergone AAA screening. These findings were consistent with the actual screening rate observed in this study. Third, our study showed that providers thought that lack of patient knowledge was also a barrier to appropriate ordering practices. Indeed, in our experience, public awareness of AAA screening is lower than that of other preventive screening measures; because it is a recent recommendation, it has not been publicized to the extent that breast and colon cancer screening have.

### Potential interventions to improve compliance

These tools include developing a physician-directed protocol that would enable nursing staff to identify eligible patients and order a screening ultrasound during the rooming process. Most providers surveyed in this study believed that such a protocol would improve screening ordering rates. A similar model for cancer screening has been found to be superior to the traditional physician-directed model [14]. In addition, surveillance systems for AAA with nurse-practitioner oversight have achieved compliance with screening guidelines as high as 98.5% over a 7-year period [15]. We did not identify any differences in screening rates according to provider gender or role. Staff physicians did not screen at greater rates than other providers, suggesting that interventions to improve screening should not be limited to specific provider groups.

From National Ambulatory Medical Care Survey data [16], we know that men have fewer office visits than women in the screening age group. For men, who may not visit a clinic for years, mailed reminders such as those successfully used to increase screening mammography rates could be employed [17]. In addition, advances in HIT can be used to boost patients' completion rates of preventive screenings. For example, with the advent of electronic patient health records, patients can track and manage their own relevant preventive or screening services [18]. Mayo Clinic recently launched such an online patient-managed personal health record system, Mayo Clinic Health Manager, that patients can use to receive reminders and recommendations tailored to their lifestyle and health status [19].

However, rather than relying on methods that place the burden on the patient to initiate scheduling of important screening for services in which the test poses no (or minimal) risk to the patient (e.g. AAA, osteoporosis, breast cancer), allied health staff can be trained to schedule screenings when the patient is due. In our study of breast cancer screening, a part-time trained appointment secretary managed the breast cancer screenings for thousands of patients [17]. In the future, HIT systems may allow patients to directly schedule these types of preventive screenings when they become eligible or due for those services, and primary care providers would need to interact with the patient only if the test results were abnormal. The importance of delivery rates of preventive services and electronic connectivity with patients is reflected in the inclusion of multiple metrics for these two health care components in the newly announced ‘meaningful use’ measures for HIT [20].

### Impact on other clinical practices

New infrastructures are needed to improve preventive services such as AAA screening. The patient-centred medical home calls for a personal physician to lead a team of individuals to provide comprehensive care for patients, including preventive services, through multiple modalities. [21,22] Examining the quality of preventive care as we have done in this study and the use of electronic systems such as our automated reminder system are important features of this model. This study further reinforces the concept of the medical home in that multiple modalities, in addition to electronic systems, are needed for providing preventive services, particularly when the visit time is a limiting factor. Additional resources discussed previously are needed to improve compliance. Similar strategies presented in this study can be employed in other clinics to identify areas of suboptimal performance and to improve the quality of care delivered to patients in primary care practices.

### Study limitations

This study had several limitations. First, it was performed over a 4-month period, so the results only approximate actual AAA screening ordering rates over longer periods. Second, ordering practices at only the initial visit during the study period were examined, and it is plausible that some patients had an ultrasound ordered on a subsequent visit. However, we found that, even if all visits during the study interval were included, only 15.5% of eligible patients had AAA screening ordered by their provider. Aside from patient refusal, other patient factors (e.g. insurance status, ethnicity, language barriers, medical comorbidities) were not assessed. Consequently, it is unknown if any of these factors contributed to the low rate of AAA screening ordering. Finally, because this study involved only a single practice site, it may not represent screening ordering rates in the rest of country.

## Conclusions

This study demonstrated low rates of AAA screening ordering for patients who met the eligibility criteria. It also showed that rates were independent of provider type or gender but were related to visit length. Because providing preventative services is often time-consuming, electronic reminder systems have often been employed in clinical practices like ours around the country. Such systems may not act as a magic bullet to this problem. A more comprehensive and multidisciplinary approach is required, as illustrated by the concept of the medical home, to improve preventive screening rates. Future studies should examine the effect of interventions (e.g. nurse-directed ordering protocol, patient-directed mailed reminder campaign, provider education measures) on AAA screening.

## Abbreviations

AAA: abdominal aortic aneurysm
USPSTF: US Preventive Services Task Force
HIT: health information technology
